# 2,4-dichlorophenoxyacetic acid-induced oxidative stress: Metabolome and membrane modifications in *Umbelopsis isabellina*, a herbicide degrader

**DOI:** 10.1371/journal.pone.0199677

**Published:** 2018-06-22

**Authors:** Przemysław Bernat, Justyna Nykiel-Szymańska, Paulina Stolarek, Mirosława Słaba, Rafał Szewczyk, Sylwia Różalska

**Affiliations:** Department of Industrial Microbiology and Biotechnology, Faculty of Biology and Environmental Protection, University of Lodz, Lodz, Poland; University of Illinois, UNITED STATES

## Abstract

The study reports the response to herbicide of the 2,4-dichlorophenoxyacetic acid (2,4-D)–degrading fungal strain *Umbelopsis isabellina*. A comparative analysis covered 41 free amino acids as well as 140 lipid species of fatty acids, phospholipids, acylglycerols, sphingolipids, and sterols. 2,4-D presence led to a decrease in fungal catalase activity, associated with a higher amount of thiobarbituric acid-reactive substances (TBARS). Damage to cells treated with the herbicide resulted in increased membrane permeability and decreased membrane fluidity. Detailed lipidomic profiling showed changes in the fatty acids composition such as an increase in the level of linoleic acid (C18:2). Moreover, an increase in the phosphatidylethanolamine/phosphatidylcholine ratio was observed. Analysis of fungal lipid profiles revealed that the presence of 2,4-D was accompanied by the accumulation of triacylglycerols, a decrease in ergosterol content, and a considerable rise in the level of sphingolipid ceramides. In the exponential phase of growth, increased levels of leucine, glycine, serine, asparagine, and hydroxyproline were found. The results obtained in our study confirmed that in the cultures of *U*. *isabellina* oxidative stress was caused by 2,4-D. The herbicide itself forced changes not only to membrane lipids but also to neutral lipids and amino acids, as the difference of tested compounds profiles between 2,4-D—containing and control samples was consequently lower as the pesticide degradation progressed. The presented findings may have a significant impact on the basic understanding of 2,4-D biodegradation and may be applied for process optimization on metabolomic and lipidomic levels.

## Introduction

Auxin-like 2,4-dichlorophenoxyacetic acid (2,4-D) is commonly used to control weeds among cereal crops such as corn and wheat [1]. However, extensive application of 2,4-D may cause toxicological problems in non-target organisms, including soil microbiota [2]. There are reports of the toxic effects of 2,4-D on the growth of soil bacteria such as *Delftia acidovorans* and *Pseudomonas putida* [2, 3, 4, 5]. Stress shock proteins have been found to be induced by 2,4-D in growing cultures of *Burkholderia* sp. K-2 –a strain isolated from contaminated soils [6]. In *Escherichia coli* cells treated with 2,4-D, a rougher cell surface and modifications of oxidative phosphorylation, the ABC transport system, and peptidoglycan biosynthesis have been observed [7]. Furthermore, the toxic effects of 2,4-D have been studied using microbial models of *Saccharomyces cerevisiae* [8, 9], which are not able to metabolize this herbicide. It seems that the liposoluble form of this toxic compound affects the spatial organization of the membrane and impairs its function as a permeability barrier [8, 9]. However, studies on the negative effects of 2,4-D on soil filamentous fungi, including those responsible for biodegradation processes, are very rarely available. In our previous study, we have described an *Umbelopsis isabellina* strain capable of degrading the 2,4-D pesticide [10]. Species of the genus *Umbelopsis* are saprobes in soil and are well-known for being capable of accumulating lipids containing γ-linolenic acid (GLA) using different media as carbon and nitrogen sources [11, 12]. Additionally, there are reports about their biotransformation potential, i.e. hydroxylation of asiatic acid, dehydroabietic, abietic, and isopimaric acids biotransformation [13, 14]. Additionally, some studies reveal their ability to degrade and reduce the toxicity of the endocrine disruptors nonylphenol, 4-tert-octylphenol and 4-cumylphenol [15]. Since 2,4-D is a membrane-active molecule, the interactions of the herbicide with lipids may play an important role in its toxicity mechanisms. Therefore, the aim of this study was to evaluate current understanding of the relationship between 2,4-D biodegradation and the composition of fungal lipids that are rich in polyunsaturated fatty acids. The present study may also provide valuable information about the promotion of oxidative stress in this herbicide-metabolizing fungus in terms of lipid composition and the concentration of free amino acids. To understand how individual lipid species from different lipid classes contribute to herbicide toxicity, we conducted a liquid chromatography–mass spectrometric (LC-MS/MS) analysis of the lipid composition of *U*. *isabellina*. We identified individual species of fatty acids, phospholipids, sphingolipids, sterols, diacylglycerols (DAGs), and triacylglycerols (TAGs). Furthermore, we measured membrane condition in terms of permeability, potential, and fluidity. In addition, we described the contribution of the antioxidant enzymes superoxide dismutase (SOD) and catalase (CAT) in the protective mechanism of the 2,4-D–degrading fungus against oxidative stress. Further, the reactive oxygen species (ROS) and reactive nitrogen species (RNS) generated within cells were detected using a confocal laser scanning microscopy (CLSM) technique.

## Materials and methods

A detailed description of the materials and methods used is available in the supplementary materials.

### Reagents

2,4-D, butylated hydroxytoluene (BHT), thiobarbituric acid, ergosterol, 1,3-dioleoyl-2-palmitoylglycerol, dioleoylglycerol, 2′, 7′-dichlorodihydrofluorescein diacetate (H_2_DCFDA), and malondialdehyde (MDA) were purchased from Sigma-Aldrich (Poznan, Poland). Phospholipid standards were purchased from Avanti Polar Lipids (Alabaster, AL, USA). Sphingolipids standards were procured from Cayman Chemical (Ann Arbor, MI, USA). All other chemicals were acquired from Avantor Performance Materials (Gliwice, Poland). Stock solutions of 2,4-D were prepared at a concentration of 5 mg mL^−1^ in ethanol.

### Strain and growth conditions

*U*. *isabellina* DSM 1414 (previously known as *Mortierella isabellina*) was purchased from the German Collection of Microorganisms and Cell Cultures (Braunschweig, Germany).

Seven-day-old spores of the *U*. *isabellina* strain from cultures on ZT agar plants were used to inoculate 20 mL Sabouraud dextrose broth medium (Difco) in 100 mL Erlenmeyer flasks [10]. The cultivation was performed on a rotary shaker (160 rpm) for 24 h at 28°C. This pre-culture was transferred to a fresh medium at the ratio 1:1 and incubated for the next 24 h. Thereafter, 2 mL of this homogenous pre-culture was introduced either into growth medium supplemented with 100 mg L^−1^ 2,4-D or into the control culture without the herbicide. All cultures were incubated at 28°C on a rotary shaker (160 rpm). The biomass was separated, and its dry weight was quantified by the method described by Bernat et al., [16].

All experiments were conducted in the exponential (24 h) and stationary (120 h) growth phases for control and 2,4-D–treated mycelium.

### 2,4-D analysis

Quantities of 2,4-D in the examined cultures were determined according to the procedure described in our previous work [10].

### Enzyme extraction and assays

The washed fresh mycelium was homogenized (1:10 w/v) in an ice-cold mortar together with 50 mM sodium phosphate buffer (pH 7) containing 1% polyvinylpyrrolidone, 10 mM sodium ascorbate, and 1 mM EDTA. After centrifugation (20 min, 20,000×*g*), the supernatant was used for the determination of antioxidant enzymatic activity [17]. The CAT activity was measured spectrophotometrically at 240 nm by a method proposed by Dhindsa et al. [18]. Moreover, the total SOD activity was determined spectrophotometrically at 540 nm according to the method described by Beauchamp and Fridovich [19]. The protein content in the tested samples was assayed using the method proposed by Bradford [20].

### Lipid extraction

Lipids of *U*. *isabellina* were extracted according to the method proposed by Folch et al. [21], with some modifications. Briefly, 100 mg fungal biomass was separated on filter paper, washed with distilled water, and transferred into 1.5 mL Eppendorf tubes containing glass beads, 0.66 mL methanol, and 0.33 mL chloroform. The homogenization process, using a ball mill (FastPrep-24, MP-Biomedicals), was conducted for 2 min. The mixture was transferred to another Eppendorf tube. To facilitate the separation of two layers, 0.2 mL of 0.9% saline was added. The lower layer was collected and evaporated under reduced pressure.

### Determination of lipid peroxidation

The degree of lipid peroxidation was measured in terms of the content of thiobarbituric acid-reactive substances (TBARS) as described by Jo and Ahn [22], with some modifications. The freshly harvested fungal biomass (500 mg) was transferred into a test Falcon tube (50 mL) with 9 mL deionized water; the mixture was homogenized with a ball mill (Retsch MM 400) for 5 min at 30 Hz; to this, BHT (7.2%, 50 μL) was added before homogenization. The fungal homogenate (1 mL) was transferred a disposable test tube (10 mL), to which 2 mL TBA–TCA solution (20 mM TBA in 15% TCA) was subsequently added. The mixture was vortexed, heated in a 95°C water bath for 30 min, cooled in a cold water bath for 10 min, and centrifuged at 2,000×*g* for 15 min. The absorbance of the supernatant was measured at 531 nm using a spectrophotometer. The value of nonspecific absorption was subtracted at 600 nm.

### Fatty acid analysis

A lipid sample, prepared according to the steps described in the above section 2.4 was diluted in 1.5 mL methanol and transferred to a screw-capped glass test tube. To this lipid solution, 0.2 mL toluene and 0.3 mL HCl solution (8.0%) were added [23]. The tube was vortexed and, then, incubated overnight at 45°C. After cooling to room temperature, 1 mL hexane and 1 mL water (deionized) were added for the extraction of fatty acid methyl esters (FAMEs). The tube was vortexed and 0.3 mL of the hexane layer was moved to the chromatographic vial.

The FAMEs analysis was conducted with an Agilent Model 7890 gas chromatograph equipped with a 5975C mass detector. With helium as a carrier gas, a capillary column HP 5 MS methyl polysiloxane (30 m × 0.25 mm i.d. × 0.25 mm ft) was applied. The temperature of the column was maintained at 60°C for 3 min, then increased to 212°C at the rate of 6°C min^−1^, followed by an increase to 245°C at the rate of 2°C min^−1^, and, finally, to 280°C at the rate of 20°C min^−1^, at which it was held for 10 min. Split injection of the injection port at 250°C was employed. Fungal fatty acids were identified by comparison with authenticated reference standards (Sigma, Supelco).

### Determination of phospholipids

A lipid sample, prepared according to the method described in the previous section, was diluted in 1 mL methanol: chloroform (4:1, v/v). The polar lipids were measured using an Agilent 1200 HPLC system (Santa Clara, CA, USA) and a 4500 Q-TRAP mass spectrometer (Sciex, Framingham, MA, USA) equipped with an ESI source. Then, 10 μL lipid extract was injected onto a Kinetex C18 column (50 mm × 2.1 mm, particle size: 5 μm; Phenomenex, Torrance, CA, USA), heated at 40°C, with the flow rate of 500 μL min^−1^. Water (A) and methanol (B) were applied as a mobile phase, with both containing 5 mM ammonium formate. The solvent gradient was initiated at 70% B and, after 0.25 min, increased to 95% B for 1 min; then, it was maintained at 95% B for 7 min before returning to the initial solvent composition over 2 min. The data analysis was conducted with Analyst v1.6.2 software (Sciex, Framingham, MA, USA).

The phospholipids were determined qualitatively according to the methods described earlier [24]. Then, using a phospholipid standard for each PL class: phosphatidic acid (PA 12:0/12:0), phosphatidylcholine (PC 14:0/14:0), phosphatidylethanolamine (PE 14:0/14:0), phosphatidylglycerol (PG 14:0/14:0), LPC (16:0) and phosphatidylinositol (PI 16:0/16:0), quantitation method was prepared (S1 Table).

### Determination of acylglycerols

Lipids were measured using the same LC–MS/MS model as for the determination of phospholipids. We prepared MRM scans including parent–daughter pairs of an acylglyceride species reflecting the loss of one fatty acid for TAGs and SIM for DAGs (S2 Table). Chromatographic separation was conducted on a C18 column (the same model as mentioned above) that was heated to 40°C. The mobile phases were water (A) and a mixture of acetonitrile:isopropyl alcohol (5:2) with 5 mM ammonium formate and 0.1% formic acid (B). The following mobile phase gradient was used: mobile phase B was increased to 100% from 35% during 4 min; after 11 min, it decreased to 35% over 2 min; the flow rate was set to 0.6 mL min^−1^.

### Analysis of sphingolipids

We extracted 100-mg biomass samples with 4 mL ethyl acetate/isopropanol/water mixture (60:30:10, v/v/v) [25]. Qualitative analysis of sphingolipids from evaporated extracts was obtained by examining their mass spectrum using a triple quad mass spectrometer QTRAP 4500 (Sciex) operating in the MRM positive ionization mode as previously described [26]. For the reversed-phase chromatographic analysis, 10 μL of the lipid extract was injected on a C18 column (the same model as mentioned above). The solvents and gradient elution were identical to that applied for the determination of phospholipids.

### Determination of ergosterol

Sterol analysis was undertaken using a QTRAP 3200 (Sciex) mass spectrometer connected to a 1200 series HPLC system. A Kinetex C18 column was used. The solvents were water and methanol, with both containing 5 mM ammonium formate. Analytes were eluted with the following gradient: 40% solvent B from 0 to 1 min, 100% solvent B from 1 to 4 min, 40% solvent B from 4.0 to 4.1 min, 40% solvent B from 4.1 to 6 min with a flow rate of 0.8 mL min^−1^. The QTRAP instrument was set to the positive ion mode, with an atmospheric pressure chemical ionization (APCI) temperature of 550°C. The monitored MRM pairs were *m/z* 379.3–69.1 and 379.3–81.3.

### Test of cell membrane condition

The membrane potential was examined using bis-(1,3-dibutylbarbituric acid)trimethine oxonol (DiBAC_4_(3)) according to a modified procedure described by Liao et. al [27].

The membrane fluidity was investigated according to the method by Kuhry et al. [28] with some modifications.

The permeability of the fungal membranes was examined according to the method described by Siewiera et al. [29].

### ROS determination with the H_2_DCFDA technique

The ROS production in the fungal biomass was determined with a cell-permeant (H_2_DCFDA; Sigma–Aldrich, Germany) by a method described previously by our team [17].

### Extraction and analysis of amino acids

We transferred 100 mg fungal biomass into Eppendorf tubes containing glass beads and 1 mL ethanol (80%) solution in water. Homogenization with FastPrep-24 (MP-Biomedicals) was conducted for 2 min. Next, the sample was centrifuged (2 min, 6,000×*g*) and 50 μL of the supernatant was diluted with deionized water. Fungal amino acid concentrations were determined in duplicates by an aTRAQ Kit for amino acid analysis of physiological fluids (Sciex) with a QTRAP 4500 (Sciex) mass spectrometer connected to an Eksigent microLC 200 (Sciex). Detailed analysis was performed according to the manufacturer’s instructions.

### Data acquisition and statistical analysis

Comparison of the control and 2,4-D–treated mycelium was performed using the mean of three independent biological replicates ± standard error of the means from individual samples. The results were estimated by ANOVA and statistical analyses were performed on three replicates of data obtained from each treatment. The significance (P<0.05) of differences was treated statistically by one-, two- or three-way ANOVA. Analysis was performed using the software STATISTICA ver. 13.0 (StatSoft).

## Results

### Effects of 2,4-D on the activity of *U*. *isabellina* antioxidant enzymes and peroxidation of fungal lipids during 2,4-D biodegradation

Our previous studies have demonstrated that *U*. *isabellina* degraded 2,4-D when grown in a synthetic medium [10]. Therefore, modifications in fungal activity were observed both for the exponential phase of growth, when most of the added xenobiotic was present in the culture (>80% of the initial content), and for the stationary phase of growth, after >70% of the added herbicide was metabolized. The CAT level decreased in the samples with 2,4-D from both the exponential and stationary growth phases. Two-way ANOVA revealed that the growth phase (F = 3442.20, P<0.001) and exposure to 2,4-D (F = 1045.09, P<0.001) significantly influenced the CAT activity. However, there was no interaction between the growth phase and the treatment with the herbicide (F = 0.87, P = 40). In contrast, the activity of SOD remained at the control level after the treatment with 2,4-D for 24 and 120 h without statistical significance (Table 1).

**Table 1:** Activity of catalase (CAT) and superoxide dismutase (SOD), and TBARS levels determined in *U*. *isabellina* biomass cultivated on Sabouraud medium with the presence or absence of 2,4-D [initial concentration 100 mg L^-1^].

Parameter	24h		120h	
	control	2,4-D	control	2,4-D
CAT activity [U mg protein^-1^]	12.37±0.11	8.11±0.29	20.5±0.21	16.02±1.14
SOD activity [U mg protein^-1^]	0.77±0.04	0.62±0.08	0.7±0.08	0.9±0.12
**TBARS [uM g^-1^]**	23.5±0.7	38.18*±0.26	36.5±2.82	43.69*±0.45

Furthermore, using H_2_DCFDA, we found that the ROS level in the fungal biomass peaked during the initial 24 h of incubation (S1 Fig). Moreover, lipid peroxidation was investigated by measuring TBARS and expressing this in terms of MDA content. The presence of the herbicide caused an approximately 1.5-fold increase in the TBARS levels for the mycelia from the exponential growth phase (Table 1). Two-way ANOVA demonstrated a significant effect of the exposure to 2,4-D (F = 109.80, P<0.001) and incubation time (F = 78.06, P<0.001) as well as their interaction effect (F = 1315.8, P<0.001) and the interaction between the treatment with the herbicide and the culture time (F = 12.81, P = 0.02) on the TBARS levels.

### Condition of the fungal membrane in the presence of 2,4-D

Using the anionic fluorophore DiBAC_4_(3), which permeates depolarized cell membranes and binds to intracellular proteins with fluorescence enhancement, a higher intensity of fluorescence was observed in 2,4-D–treated cells harvested from the exponential phase of growth. However, in the stationary phase of growth, no significant difference (P = 0.09) was found between the cells exposed to the xenobiotic and the control cells (Table 2).

**Table 2:** Effect of 2,4-D on the fluorescence intensity of 1,6-diphenyl-l,3,5-hexatriene (DPH), fluorophore DiBAC_4_ and propidium iodide fluorescence in fungal biomass in the exponential and stationary phases of growth.

Parameter	Exponential phase		Stationary phase	
	control	2,4-D	control	2,4-D
DPH	365.2 ±71.2	878.3 ±104.7	5149.9 ±455.4	3717.8 ±305.5
DiBAC_4_(3_)_	662.2 ±70.7	2066.3 ±373.0	6676.9 ±573.5	6223.6 ±50.8
Propidium iodidie	253.5 ±55.0	949.0±82.4	918.7 ±89.2	1366.5 ±134.0

We observed the effect of the pesticide on the membrane integrity of the fungal cell. Fungal cultures from the exponential and stationary phases of growth were stained with propidium iodide. The dye, after passing through the damaged cell membrane and binding to the DNA, was detected using CLSM and a spectrofluorometer. In the presence of the toxic compound, more than 3- and 1.5-fold increases in membrane permeability were observed in the control background in the exponential and stationary phases of growth, respectively. Two-way ANOVA showed a significant effect of the growth phases (F = 97.78, P<0.001) and exposure to the herbicide (F = 109.06, P<0.001) on the permeability of the *U*. *isabellina* membrane. However, there was no significant effect of the interaction between the growth phase and the exposure to 2,4-D (F = 5.12, P<0.053). To investigate the possible influence of 2,4-D on membrane fluidity, we conducted measurements on cells harvested in the exponential and stationary growth phases. As illustrated in Table 2, fluidity in cells from mycelium exposed to 2,4-D demonstrated higher values than control samples in the exponential phase of growth as well as significantly lower levels in the stationary phase. Two-way analysis of variance revealed a significant effect of the growth phase and the treatment with 2,4-D (F = 15.64, P = 0.004) on the fluidity of the *U*. *isabellina* membrane.

### Influence of 2,4-D on the fatty acid profile of *U*. *isabellina*

The effect of 2,4-D on the composition of the whole-cell–derived fatty acids of *U*. *isabellina* (Table 3) was observed. The gas chromatography mass spectrometry (GC/MS) investigation showed that *U*. *isabellina* was dominated by three types of fatty acids: saturated (16:0, 18:0), monounsaturated (16:1, 18:1), and polyunsaturated (18:2, 18:3). Other fatty acids (14:0 and 20:0) were found in small amounts.

**Table 3:** Fatty acid contents (%) of *U*. *isabellina* during cultivation on Sabouraud medium^a^.

Fatty acid	Exponential phase		Stationary phase	
	Control	2,4-D	Control	2,4-D
C14:0	0.94	0.70	0.92	0.86
C16:1	1.17	0.81	1.71	1.5
C16:0	26.51	25.45	22.95	20.29
C18:3	9.34	6.87	9.98	8.18
C18:2	9.49	20.17	10.98	15.55
C18:1	45.84	38.89	47.26	45.67
C18:0	6.63	6.97	5.74	7.47
C20:0	0.08	0.10	0.46	0.48

^a^Values are the means of triplicates that varied between 2 and 8%.

*U*. *isabellina* degraded 2,4-D in the course of its growth. Therefore, fatty acid analysis was conducted for the exponential and stationary phases of growth (Table 3). Three-way ANOVA indicated that the interaction between the growth phase, the treatment with 2,4-D and different types of fatty acids had no effect on the composition of fungal fatty acids (F = 2.05, P = 0.06). However, interactions between the types of fatty acids and the growth phase (F = 8.1, P<0.001) and interactions between the types of fatty acids and exposure to 2,4-D (F = 9.54, P<0.001) significantly influenced the composition of *U*. *isabellina* fatty acids. As a result of the treatment with the pesticide, the contents of oleic and linoleic fatty acids in the fungus changed significantly (at the end of culture, 45.67% and 15.55%, in comparison with the control, 47.26% and 10.98%, respectively). Moreover, the ratio of unsaturated to saturated fatty acids increased from 2.01 to 2.43 during 2,4-D degradation for the exponential and stationary phases of growth, respectively.

### Phospholipids

**Fig 1:**
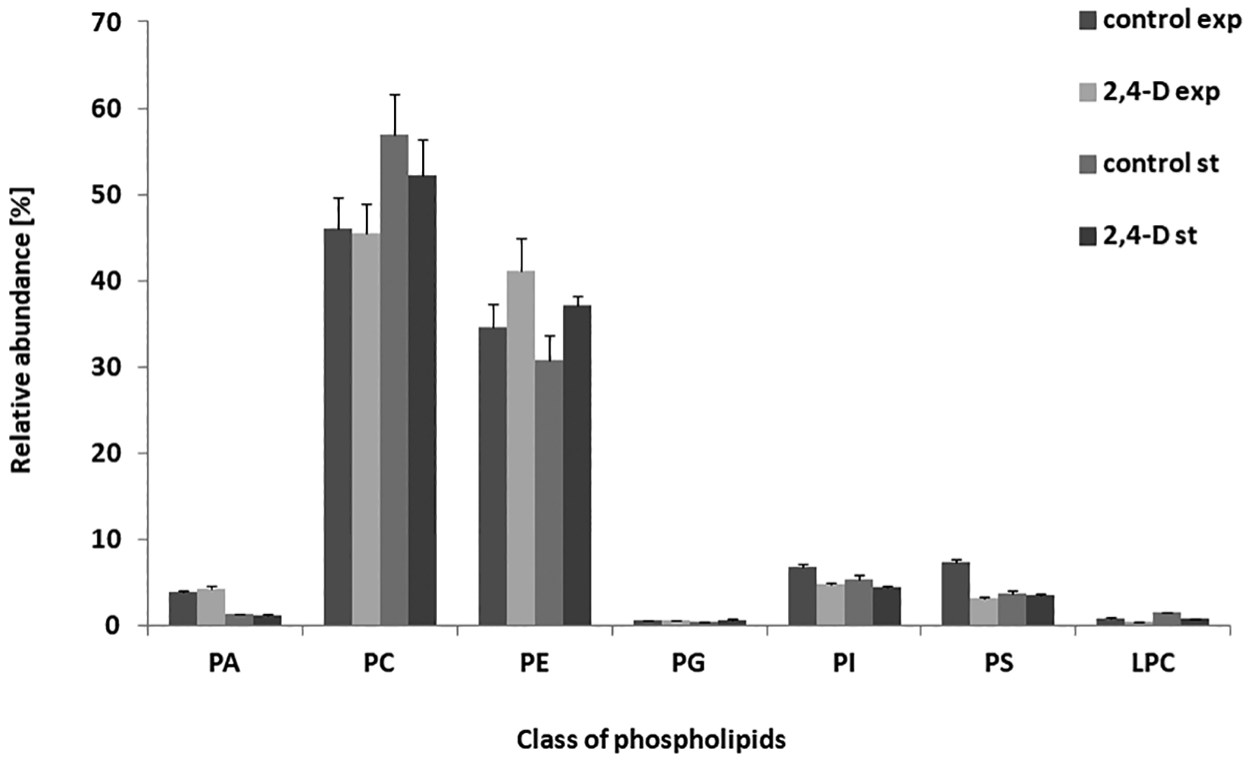
Comparison of phospholipid composition of *U*. *isabellina* from the exponential and stationary phases of growth exposed to 2.4-D. PA, phosphatidic acid; PE, phosphatidylethanolamine; PC, phosphatidylcholine; PG, phosphatidylglycerol; PI, phosphatidylinositol; PS, phosphatidylserine; LPC, lysophosphatidylcholine.

In the next stage of the study, phospholipids—the main lipid constituent of the membranes—were investigated. Using the LC–MS/MS procedure, we identified 93 species of *U*. *isabellina* phospholipids, including the following species: PA, PC, lysophosphatidylcholine (LPC), PE, PI, PG and PS in the numbers of 13, 21, 6, 20, 17, 3, and 13, respectively (S1 Table). Among these, PC was the predominant phospholipid and constituted 46–57% of the total cell phospholipids in the control sample, followed by PE, which constituted 31–34% of the total cell phospholipids. Levels of the other species of phospholipids were less than 7.3% (Fig 1). Three-way ANOVA revealed that the interaction between the growth phase, the species of PLs and the exposure to 2,4-D had no effect on the content of *U*. *isabellina* phospholipids (F = 0.96, P = 0.46). However, interactions between the species of phospholipids and the growth phase (F = 16.92, P<0.001) and interactions between the species of phospholipids and the treatment with 2,4-D (F = 7.27, P<0.001) significantly influenced the content of *U*. *isabellina* phospholipids.

On comparing samples from the herbicide-supplemented medium with the mycelium in the control, we found that the strain exposed to 2,4-D at the exponential phase of growth had significantly higher levels of PE (P<0.01; in contrast to the control samples; Fig 1). In the stationary phase of growth, we found differences between the samples.

Modifications in the lipid profile were observed for other classes of phospholipids. 2,4-D reduced the percentages of LPC, PI, and PS, and slightly increased the amount of PA in the exponential phase of growth.

**Fig 2:**
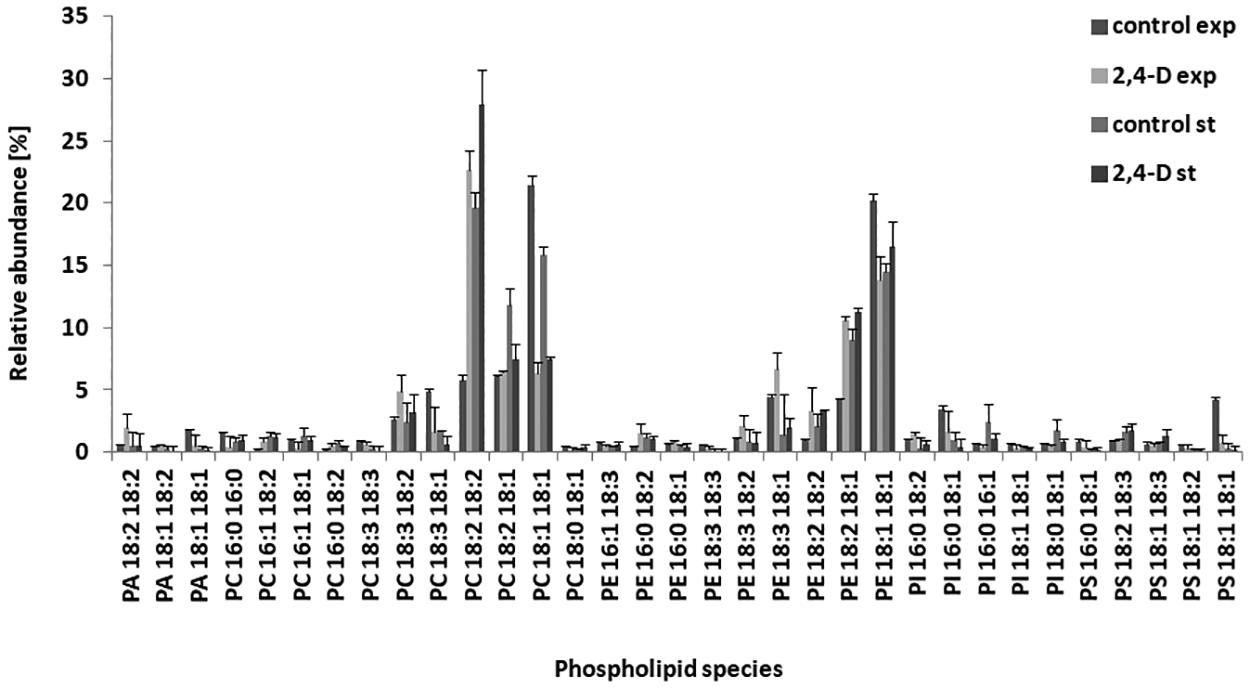
Relative abundance of dominant PL species of *U*. *isabellina* from the exponential and stationary phases of growth exposed to 2.4-D. PA, phosphatidic acid; PE, phosphatidylethanolamine; PC, phosphatidylcholine; PI, phosphatidylinositol; PS, phosphatidylserine; LPC, lysophosphatidylcholine.

The GC/MS analysis revealed that C18:1 was the main fungal fatty acid. Therefore, PCs at *m/z* 828.5 (18:1 18:2), *m/z* 830.5 (18:1 18:1), and PEs and *m/z* 742.5 (18:1 18:1) predominated in all the cultures. Moreover, in mycelia samples treated with 2,4-D and control samples (stationary phase of growth), PCs at *m/z* 826.5 (18:2 18:2) were also clearly visible (Fig 2).

### Acylglycerols

**Fig 3:**
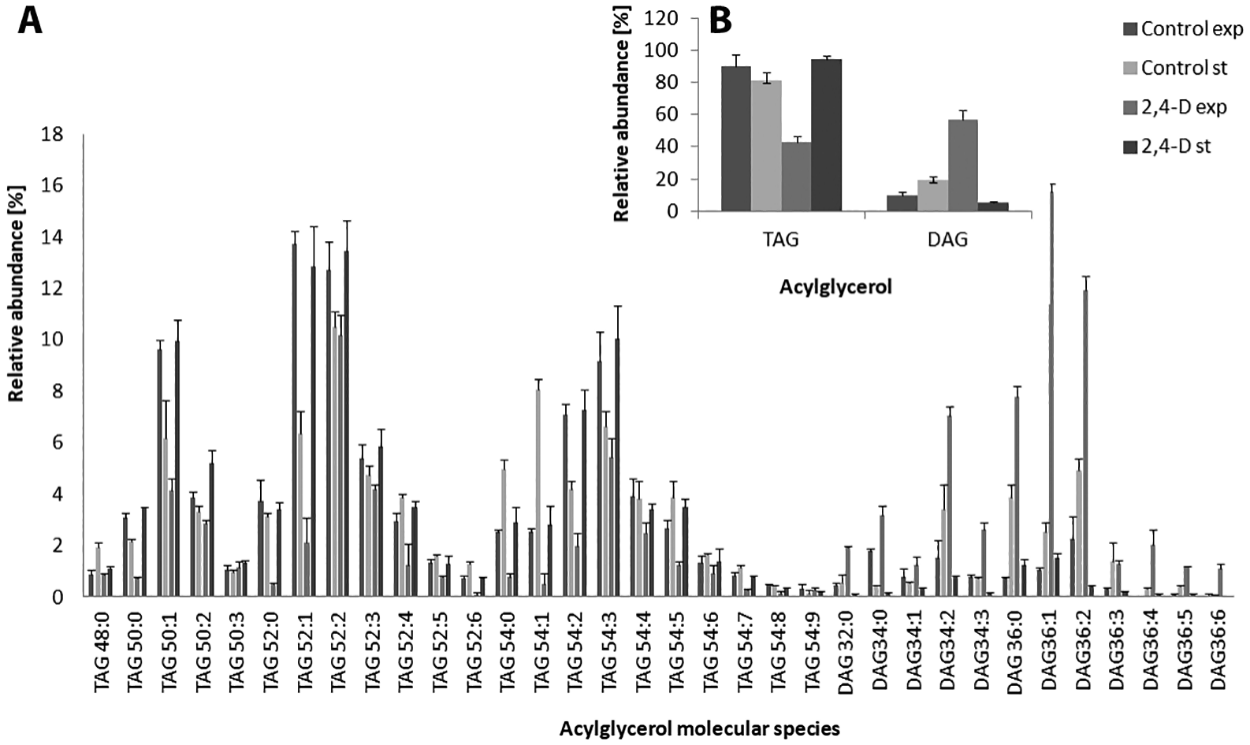
Acylglycerol species (A) and percentage distribution (B) of *U*. *isabellina* exposed to 2.4-D. TAG, triacylglycerol; DAG, diacylglycerol; Exp, exponential phase of growth; St, stationary phase of growth.

Using the LC–MS/MS, 22 species of TAG (S2 Table) and 12 of DAG were identified (Fig 3). Notably, NH_4_^+^ adducts of TAGs at *m/z* 876.8 (52:2), *m/z* 902.8 (54:3), and *m/z* 878.8 (52:1) predominated in the strain, indicating that the major molecular species of TAG were 16:0/18:1/18:1, 18:1/18:1/18:1 and 16:1/18:0/18:0. For DAG, the major species were 18:0/18:1 and 18:1/18:1. Distinct differences between the acylglycerol profile and the control sample were observed for the biomass from the exponential phase of growth (P<0.001). In this period, a strong increase in the DAG level was found (Fig 3B). Three-way ANOVA revealed that the interaction between the growth phase, the acylglyceride species and exposure to 2,4-D significantly influenced the acylglycerol content in *U*. *isabellina* (F = 39.60, P<0.001).

### Sphingolipids

**Fig 4:**
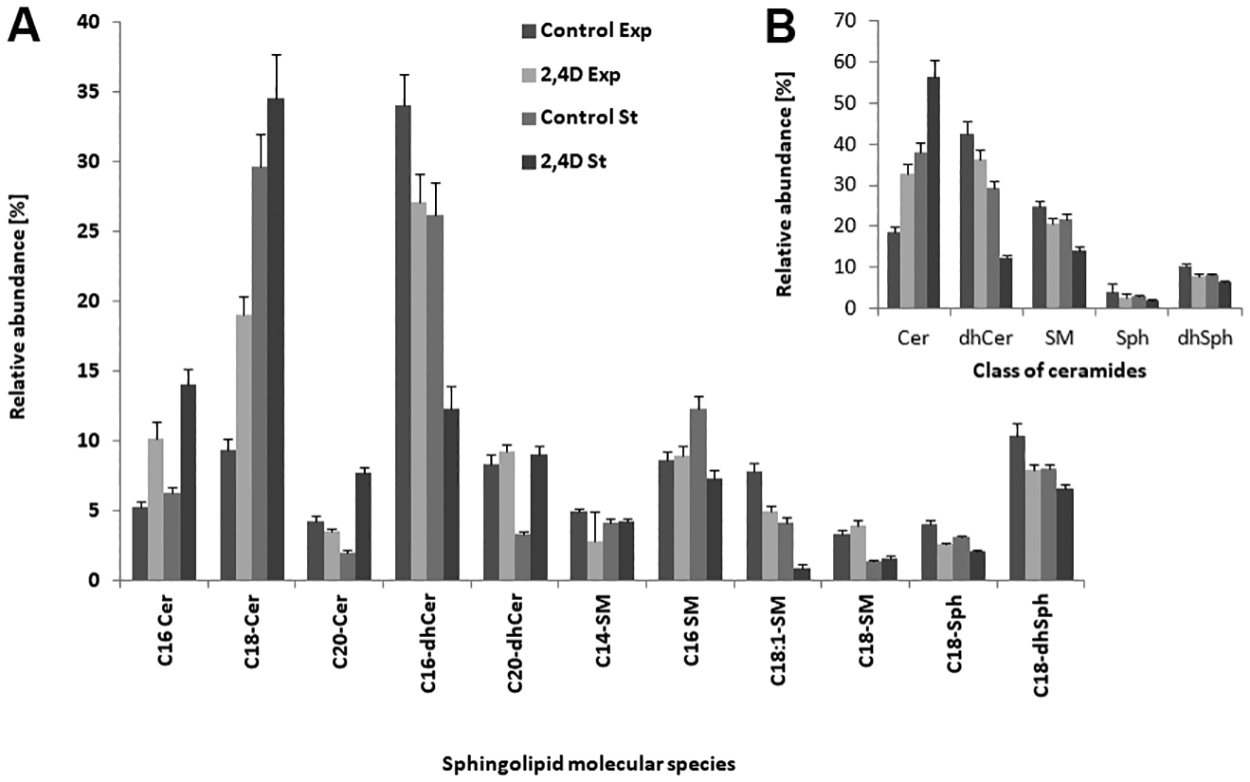
Sphingolipid species (A) and percentage distribution (B) are shown. Cer, ceramide; dhCer, dihydroceramide; Sph, sphingosine; dhSph, dihydrosphingosine; Exp, exponential phase of growth; St, stationary phase of growth.

*U*. *isabellina* was found to produce species of ceramide, dihydroceramide (dhCer), sphingosine (Sph), dihydro-sphingosine (dhSph), and sphingomyelin (SM) with long C14, C16, and very long C18 chains (Fig 4A). Of the total sphingolipid pool, ceramides and dihydroceramide dominated in fungal samples, constituting 60–77% of the total sphingolipids (Fig 4B). Three-way ANOVA indicated that the interaction between the growth phase, 2,4-D presence and sphingolipid species significantly influenced the composition of fungal sphingolipids (F = 30.55, P<0.001). The composition of the analyzed sphingolipids also varied significantly between the phases of growth (F = 1367.25, P<0.001). The dhCers were the most abundant species in the exponential phase, whereas ceramides dominated in the stationary phase. Moreover, the ceramide and dhCer contents were found to be higher in 2,4-D–treated samples compared to the control. The overall Sph and dhSph contents did not vary significantly between the samples, except for the fact that Sph and dhSph were 1.2- to 2-fold higher in the exponential phase of growth for control mycelium when compared to other samples. The SM contents ranged between 13% and 24% between cultures, and were significantly higher in the control compared to 2,4-D–exposed mycelium (Fig 4B). dhCerC16 (*m/z* 540.8) was the major dhCer species, and was most abundant in the control. Desaturation of dhCer formed ceramide structures. Among the ceramides, ceramide species C18 (*m/z* 566.4) was dominant, with levels significantly higher in the xenobiotic-exposed biomass.

### Ergosterol

A significant change in the level of ergosterol was observed while comparing the samples with added 2,4-D against the control (P<0.001). In the exponential phase of growth, the amount of the sterol was 2-fold lower in the 2,4-D–supplemented biomass (1.91 mg g dw^-1^ ±0.12 and 0.94 mg g dw^-1^ ±0.12, for control and 2,4-D, respectively). However, during the following days of incubation, the differences between the amounts of the sterol in both types of culture were less significant (3.92 mg g dw^-1^ ±0.41 and 2.57 mg g dw^-1^ ±0.2, for control and 2,4-D, respectively).

### Fungal amino acids

**Fig 5:**
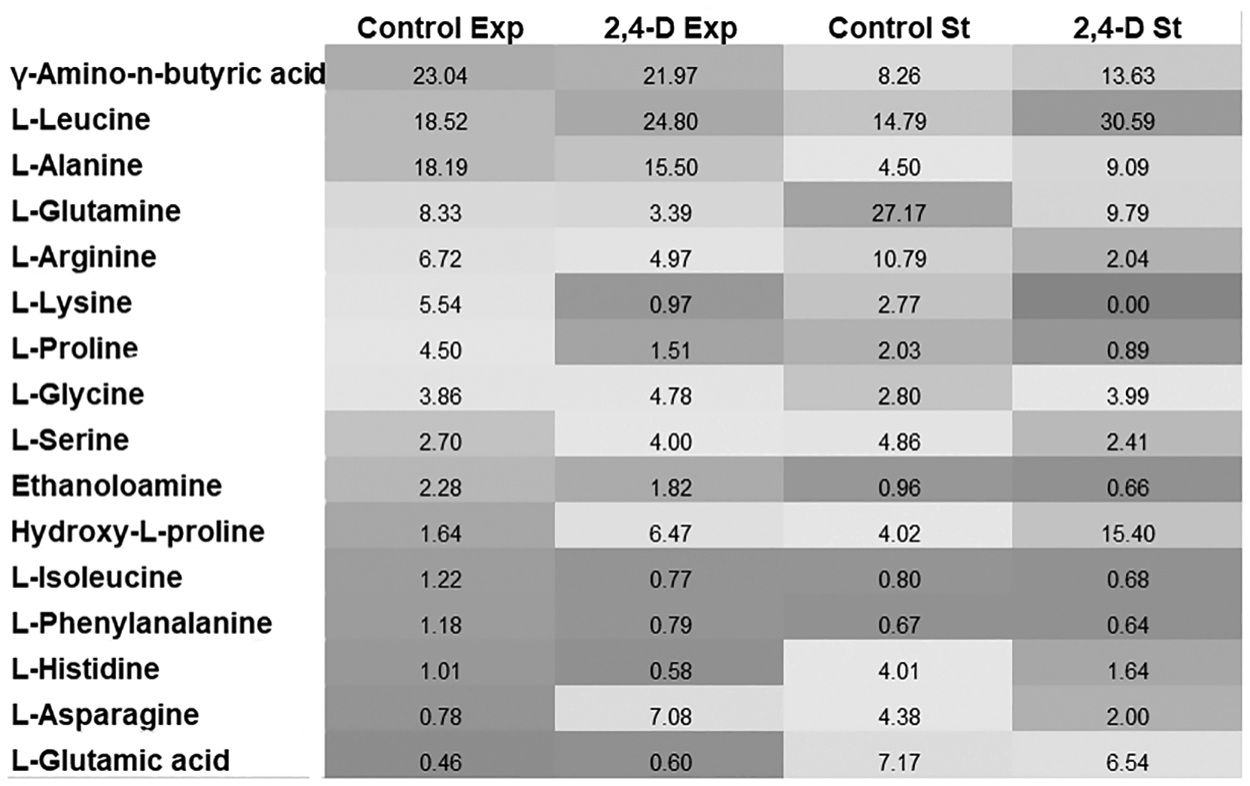
Relative amino acid concentration distribution of *U*. *isabellina* exposed to 2.4-D. (calculated as a percentage of the cumulative amount). Exp, exponential phase of growth; St, stationary phase of growth.

The influence of 2,4-D on 41 amino acids extracted from the fungal biomass was determined (Fig 5). In the biomass treated with 2,4-D, levels of most amino acids diminished. Herbicide stress increased the levels of leucine, glycine, serine, asparagine, and hydroxyproline in the exponential phase of growth. Of these, only hydroxyproline had higher levels in the stationary phase of growth.

## Discussion

The mechanism of the toxic action of phenoxy herbicides, including 2,4-D and its metabolites, toward weeds is associated with the generation of ROS and lipid peroxidation [7, 30, 31]. Pesticides adversely affect the appropriate functioning of the antioxidant system. However, the mechanism by which this pesticide can interact with the membrane and disturb lipid metabolism has still not been elucidated.

The higher TBARS level found in the *U*. *isabellina* biomass from 2,4-D cultures confirmed the induction of oxidative stress. This phenomenon results from a disturbance between the generation of ROS and their removal by the antioxidant defense system. According to Busi et al. [31], in susceptible plants treated with 2,4-D, production of H_2_O_2_ and reactive oxygen species leads to plant death.

The antioxidant enzymes CAT and SOD are important members of the first line of cell defense against oxidative stress and are well-defined biomarkers for establishing the profile of oxidative stress in organisms [32]. However, the increased ROS and RNS levels did not increase the activity of the components of the antioxidant enzyme systems in *U*. *isabellina*. A decrease in the CAT levels has also been observed for *Aspergillus niger* against an excess of Cd(II) ions [33]. It seems that an alternative mechanism of protection against H_2_O_2_ occurred in *U*. *isabellina*.

Lipid peroxidation can be defined as oxidative degradation of fatty acids containing double bonds. Among lipid fractions, phospholipids are reported to be more susceptible than other lipids [34], probably because they are integral membrane components and are available to the radicals formed in the membrane lipids. Because of the lipophilicity of 2,4-D, the fungal membrane could be a target site for its action. An assessment of membrane permeability, potential, and fluidity plays an important role in analyzing the mode of action of membrane-targeting compounds. Therefore, in this study, confocal microscopic and spectrofluorometric techniques were applied. In the exponential phase of growth, a strong increase in membrane fluidity was found in 2,4-D–treated cells. However, the 2.5-fold decrease in this parameter observed in the stationary phase of growth can be explained as a result of the high structural disruption of the bilayer hydrophobic region caused by the incorporation of 2,4-D. Decreased cell membrane fluidity triggered by higher concentrations of saturated fatty acids in the presence of 2,4-D has also been observed in *S*. *cerevisiae* cells [9]. Damage to those cells treated with the pesticide resulted in increased membrane permeability. An increase in DiBAC_4_(3) fluorescence in comparison to control cells was observed in 2,4-D–treated cells in the exponential phase of growth. As DiBAC_4_(3) enters depolarized cells, it seems that the pesticide increases depolarization of fungal membranes in the exponential phase [35].

Fatty acids are a key constituent of lipids; therefore, the influence of 2,4-D on their composition was investigated. The presence of C16:0, C16:1, C18:0, C18:1, C18:2 and C18:3 fatty acids in *U*. *isabellina* has also been reported by others [36, 37].

Because, the composition of fatty acids in fungal biomass can be influenced by the C/N ratio imposed (e.g. at low C/N media the fatty acid profiles of *Zygomycetes* are slightly more unsaturated) [11] and the carbon source used, the detailed fatty acids profiles could be difficult to compare. Strains of *U*. *isabellina* were cultivated e.g. on cheese-whey, glucose, xylose and it was observed that the fatty acids composition slightly changed [11,12]. In the present study Sabouraud medium, contains peptone providing nitrogen and dextrose as a carbon source was applied. However, oleic acid dominated (C18:1) in the fatty acids profile of the examined fungal strain and *U*. *isabellina* strains described by others [11, 36].

Desaturation ratios of *U*. *isabellina* lipids showed that, in both phases of growth, the decreased ratio of C18:3 to C18:2 might suggest the inhibition of Δ^6^ desaturase activity in the presence of 2,4-D. In contrast, Δ^12^ desaturation, converting oleic acid (C18:1) to linoleic acid (C18:2), proceeded considerably more efficiently in the fungal mycelium exposed to the herbicide. In 2,4-D–adapted *S*. *cerevisiae*, the decreased transcription of the *OLE1* gene encoding the Δ9 fatty acid desaturase suggested that yeast adaptation to the herbicide involved the enhancement of the ratio of saturated to monounsaturated fatty acids through reduced OLE1 expression [9]. However, polyunsaturated C18:3 fatty acid was most susceptible to free radical damage caused during lipid peroxidation and, therefore, its level was decreased. A similar phenomenon describing a significant increase in saturated fatty acids and a decrease in monounsaturated fatty acids and polyunsaturated fatty acids levels has been found in *Cunnighamella elegans* cells treated with tributyltin (TBT) [24].

A schematic illustration of the lipid metabolism in *U*. *isabellina* is presented in S2 Fig PC and PE are the main phospholipids that build biological membranes. Both play a key role in membrane integrity and in the maintenance of its function. PC is involved in stabilizing the membrane, whereas PE forms non-bilayer hexagonal phases [38]. The changes in the PC/PE ratio (from 1.84 to 1.4 for control and 2,4-D–treated mycelium, respectively, collected from the stationary phase of growth) indicate a significant influence of 2,4-D on the composition of the fungal membrane. In another oleaginous fungus *C*. *echinulata*, a 2-fold decrease in the PC/PE ratio was observed after exposition to carvedilol, a beta-blocker [39].

PS serves as a precursor in the syntheses of PE and PC [40]. In yeast cells, PS can be synthesized by the PS synthase reaction. The activity of PS synthase peaked faster in the medium without the pesticide than in the 2,4-D–supplemented medium and led to a higher PS level in control medium.

In fungal cells exposed to the herbicide, a decrease in the quantity of phospholipid species containing two C18:3 fatty acids, PC 18:3/18:3 and PE 18:3/18:3, was observed. A similar phenomenon has been noticed in the *C*. *elegans* cells exposed to highly lipophilic TBT [24]. This phenomenon can be explained by the fact that the formation of fatty acid radicals is easier with increasing unsaturation [41].

Acylglycerols play an important role in fungal lipid metabolism and their synthesis is strictly associated with phospholipid metabolism. Phosphatidic acid is dephosphorylated to DAG, which then serves as a precursor for TAG. Moreover, DAG is a major precursor for the glycerophospholipids PC and PE [28]. TAG is a storage molecule in fungi, which quantitatively dominates among oleaginous fungal strains such as *C*. *echinulata* or *U*. *isabellina* [36, 42, 43, 44]. This fact could probably explain the higher level of TAG in the biomass from the stationary phase of growth. In the exponential phase of growth, there was an increase in DAG, which is the precursor to all of the phospholipids that constitute the fungal cell membrane. The obtained results for *U*. *isabellina* lipids revealed that the presence of 2,4-D was accompanied by the accumulation of PE and TAG. In the study of the symbiosis of the fungus *Rhizopus microsporus* and its *Burkholderia* endobacteria, such accumulation has been observed in the fungus during symbiosis with the bacteria [42]. Moreover, it seems that the TAG fraction was less enriched in polyunsaturated fatty acids, especially γ-linolenic (GLA), than phospholipids. A similar observation was also made by Chatzifragkou et al. [45] for *M*. *isabellina* cultivated on sugar-based media or *Rhodosporidium toruloides* on waste glycerol-based media [46].

Sphingolipids are a component of the plasma and intracellular organelle membranes [47]. Their core structure is provided by a long-chain amino alcohol, commonly trans-1,3-dihydroxy-2-amino-4-octadecene, sphingosine [48]. In the *U*. *isabellina* cells, the level of these lipids was elevated in the herbicide presence. This is in agreement with some studies suggesting that ceramides are essential for mediating many stress responses. Wells et al. [49] showed that sphingolipid-deficient strains of *S*. *cerevisiae* were unable to resist heat shock. Sphingolipids have been described as a class of lipids which decrease membrane permeability [50, 51]. Moreover, the function of sterols and sphingolipids in cells is strictly correlated [46]. Ergosterol plays an important role in the regulation of fungal membrane fluidity and protein folding [48]. Sterols are responsible for the membrane structure, function, and fluidity, and are often involved in stress resistance (i.e. ethanol in yeast) [50]. In the present study, a 2-fold decrease was found in terms of the amount of ergosterol from *U*. *isabellina* cultured with the herbicide compared to the situation in control cells. Furthermore, it was observed that the ergosterol concentration increased during fungal growth. The accumulation of ergosterol was in accord with the results reported for *C*. *echinulata* grown on tomato waste hydrolysate [52]. Overall, it cannot be excluded that the observed decrease in the level of ergosterol in the herbicide presence may have been compensated by the increase in sphingolipid levels.

Amino acids play significant roles in antioxidant defense in eukaryotic cells during abiotic stress [53]. In the examined fungal samples, an increased level of hydroxyproline, a hydroxylated product of proline, was observed. Hydroxyproline has also been identified in another zygomycete, *Cunninghamella blakesleeana*, on treatment with copper [54]. A massive accumulation of asparagine was also noticed in the fungal biomass during the exponential phase of growth. According to Halford et al. [55], the amount of free asparagine increased in plants as a result of the inhibition of protein synthesis or through direct effects on asparagine metabolism. Furthermore, in the exponential phase of growth, a slight accumulation of leucine, glycine, and serine was reported, in contrast to the control biomass. Increased levels of these amino acids have also been reported in plants subjected to salt stress [56].

### Conclusion

The present study demonstrates that 2,4-D significantly disturbs lipid and amino acid metabolisms in *U*. *isabellina*. Although *U*. *isabellina* has been widely described, to the best of our knowledge, this is the first report describing lipid classes and amino acids, as well as membrane condition under the influence of an exogenous stress factor. The presence of the herbicide influenced the overall amino acid concentrations, fatty acid profiles, and the lipid class content in the fungal cells. Furthermore, we cannot ignore the fact that the herbicide modified the desaturation activity and disturbed membrane homeostasis. Moreover, 2,4-D toxicity was observed toward membrane lipids as well as storage lipids. The obtained data also revealed increased levels of TBARS, asparagine, and hydroxyproline in 2,4-D–treated *U*. *isabellina*. This was the result of oxidative stress caused by the presence of the herbicide itself, as the changes were correlated with the pesticide degradation. The intracellular mechanisms of biodegradation of herbicides such as 2,4-D are still poorly described, especially in the case of fungal degraders. The presented data may have a significant impact on the general understanding of these processes and may act as a basis for the optimization of biodegradation on metabolomic and lipidomic levels.

## Supporting information

S1 Appendix A detailed description of the materials and methods used in this study. (PDF)

S1 Table Multiple reaction monitoring (MRM) transitions for phospholipids identified in *U*. *isabellina*. (PDF)

S2 Table Multiple reaction monitoring (MRM) transitions for triacylglycerols (TAGs) identified in *U*. *isabellina*. (PDF)

S1 Fig ROS (measured with H_2_DCFDA) generated in *U*. *isabelina* cells incubated with 2,4-D at 24h (a) of incubation in comparison to 24h (b) controls without toxic compound. (DOCX)

S2 Fig Hypothetical representation of the lipid metabolism in *U*. *isabellina*. (PDF)

S3 Fig MS/MS spectra of selected lipids. (PDF)
