# Supplementary material for: 2,4-dichlorophenoxyacetic acid-induced oxidative stress: Metabolome and membrane modifications in Umbelopsis isabellina, a herbicide degrader
Source: PLoS One. 2018 Jun 22;13(6):e0199677. doi: 10.1371/journal.pone.0199677 (PMC6014680; doi:10.1371/journal.pone.0199677)
Supplement: S1 Appendix — (PDF) [file pone.0199677.s001.pdf]

Materials and methods

A detailed description of the materials and methods used in this study.

Reagents

2,4-D, butylated hydroxytoluene (BHT), thiobarbituric acid, ergosterol, 1,3-dioleoyl-2-palmitoylglycerol, dioleoylglycerol, 2′, 7′-dichlorodihydrofluorescein diacetate (H_2DCFDA), and malondialdehyde (MDA) were purchased from Sigma-Aldrich (Poznan, Poland). 1,2-Dimyristoyl-sn-glycero-3-phospho-rac-(1-glycerol) (sodium salt; 14:0/14:0 PG); 1,2-dilauroyl-sn-glycero-3-phosphoethanolamine (12:0/12:0 PE); 1,2-dimyristoyl-sn-glycero-3-phosphocholine (14:0/14:0 PC); 1,2-dipalmitoyl-sn-glycero-3-phospho-(1′-myo-inositol) ammonium salt (16:0 PI); 1,2-dimyristoyl-sn-glycero-3-phospho-L-serine sodium salt (14:0/14:0 PS), and 1,2-dimyristoyl-sn-glycero-3-phosphate (sodium salt; 14:0/14:0 PA) were purchased from Avanti Polar Lipids (Alabaster, AL, USA). *N*-palmitoyl-D-erythro-sphingosine (C16Cer), *N*-palmitoyl-D-erythro-sphingosylphosphorylcholine (C16 SM), and D-erythro-sphingosine C-18 (Sph) were procured from Cayman Chemical (Ann Arbor, MI, USA). All other chemicals were acquired from Avantor Performance Materials (Gliwice, Poland). All chemicals were high-purity-grade reagents. Stock solutions of 2,4-D were prepared at a concentration of 5 mg mL^{-1} in ethanol.

Strain and growth conditions

U. isabellina DSM 1414 (previously known as *Mortierella isabellina*) was purchased from the German Collection of Microorganisms and Cell Cultures (Braunschweig, Germany).

Seven-day-old spores of the *U. isabellina* strain from cultures on ZT agar plants were used to inoculate 20 mL Sabouraud dextrose broth medium (Difco) in 100 mL Erlenmeyer flasks [10]. The cultivation was performed on a rotary shaker (160 rpm) for 24 h at 28°C. This pre-culture was transferred to a fresh medium at the ratio 1:1 and incubated for the next 24 h.

Thereafter, 2 mL of this homogenous pre-culture was introduced either into growth medium supplemented with 100 mg L^{-1} 2,4-D or into the control culture without the herbicide. All cultures were incubated at 28°C on a rotary shaker (160 rpm). The biomass was separated, and its dry weight was quantified by the method described by Bernat et al., [16].

All experiments were conducted in the exponential (24 h) and stationary (120 h) growth phases for control and 2,4-D–treated mycelium.

2,4-D analysis

Quantities of 2,4-D in the examined cultures were determined according to the procedure described in our previous work [10].

Enzyme extraction and assays

The washed fresh mycelium was homogenized (1:10 w/v) in an ice-cold mortar together with 50 mM sodium phosphate buffer (pH 7) containing 1% polyvinylpyrrolidone, 10 mM sodium ascorbate, and 1 mM EDTA. After centrifugation (20 min, 20,000×*g*), the supernatant was used for the determination of antioxidant enzymatic activity [17]. The CAT activity was measured spectrophotometrically at 240 nm by a method proposed by Dhindsa et al. [18]. Moreover, the total SOD activity was determined spectrophotometrically at 540 nm according to the method described by Beauchamp and Fridovich [19]. Enzymatic activity (U mg $protein^{-1}$) was examined by measuring the ability of the enzyme to inhibit the photochemical reduction of nitrotetrazolium blue chloride. The protein content in the tested samples was assayed using the method proposed by Bradford [20], in which a standard curve was drawn by using values for bovine serum albumin.

Lipid extraction

Phospholipids of *U. isabellina* were extracted according to the method proposed by Folch et al. [21], with some modifications. Briefly, 100 mg fungal biomass was separated on filter paper, washed with distilled water, and transferred into 1.5 mL Eppendorf tubes containing glass beads, 0.66 mL methanol, and 0.33 mL chloroform. The homogenization process, using a ball mill (FastPrep-24, MP-Biomedicals), was conducted for 2 min. Next, the sample was centrifuged (2 min, 6,000×*g*). The mixture was transferred to another Eppendorf tube. To facilitate the separation of two layers, 0.2 mL of 0.9% saline was added. The lower layer was collected and evaporated under reduced pressure.

Determination of lipid peroxidation

The degree of lipid peroxidation was measured in terms of the content of thiobarbituric acid-reactive substances (TBARS) as described by Jo and Ahn [22], with some modifications. The freshly harvested fungal biomass (500 mg) was transferred into a test Falcon tube (50 mL) with 9 mL deionized water; the mixture was homogenized with a ball mill (Retsch MM 400) for 5 min at 30 Hz; to this, BHT (7.2%, 50 µL) was added before homogenization. The fungal homogenate (1 mL) was transferred a disposable test tube (10 mL), to which 2 mL TBA–TCA solution (20 mM TBA in 15% TCA) was subsequently added. The mixture was vortexed, heated in a 95°C water bath for 30 min, cooled in a cold water bath for 10 min, and centrifuged at 2,000×*g* for 15 min. The absorbance of the supernatant was measured at 531 nm using a spectrophotometer. The value of nonspecific absorption was subtracted at 600 nm. The concentration of TBARS was estimated by referring to a standard MDA solution.

Fatty acid analysis

A lipid sample, prepared according to the steps described in the above section 2.4 was diluted in 1.5 mL methanol and transferred to a screw-capped glass test tube. To this lipid solution, 0.2 mL toluene and 0.3 mL HCl solution (8.0%) were added [23]. The tube was vortexed and, then, incubated overnight at 45°C. After cooling to room temperature, 1 mL hexane and 1 mL water (deionized) were added for the extraction of fatty acid methyl esters (FAMEs). The tube was vortexed, 0.3 mL of the hexane layer was moved to the chromatographic vial, and 1.6 μL of the extracted samples were analyzed.

The FAMEs analysis was conducted with an Agilent Model 7890 gas chromatograph equipped with a 5975C mass detector. With helium as a carrier gas, a capillary column HP 5 MS methyl polysiloxane (30 m × 0.25 mm i.d. × 0.25 mm ft) was applied. The temperature of the column was maintained at 60°C for 3 min, then increased to 212°C at the rate of 6°C min^{-1}, followed by an increase to 245°C at the rate of 2°C min^{-1}, and, finally, to 280°C at the rate of 20°C min^{-1}, at which it was held for 10 min. Split injection of the injection port at 250°C was employed. Fungal fatty acids were identified by comparison with authenticated reference standards (Sigma, Supelco) or by their mass spectra, and the results were expressed as a percentage of the total amount of fatty acids.

Determination of phospholipids

A lipid sample, prepared according to the method described in the previous section, was diluted in 1 mL methanol: chloroform (4:1, v/v). The polar lipids were measured using an Agilent 1200 HPLC system (Santa Clara, CA, USA) and a 4500 Q-TRAP mass spectrometer (Sciex, Framingham, MA, USA) equipped with an ESI source. Then, 10 μL lipid extract was injected onto a Kinetex C18 column (50 mm × 2.1 mm, particle size: 5 μm; Phenomenex, Torrance, CA, USA), heated at 40°C, with the flow rate of 500 μL min^{-1}. Water (A) and methanol (B) were

applied as a mobile phase, with both containing 5 mM ammonium formate. The solvent gradient was initiated at 70% B and, after 0.25 min, increased to 95% B for 1 min; then, it was maintained at 95% B for 7 min before returning to the initial solvent composition over 2 min. The following instrumental settings were applied: spray voltage −4500 V, curtain gas 25, nebulizer gas 60, auxiliary gas 50, and ion source temperature 600°C. The data analysis was conducted with Analyst™ v1.6.2 software (Sciex, Framingham, MA, USA).

Two approaches – targeted and untargeted – were applied to identify phospholipids. The untargeted approach was performed with a precursor ion scanning (precursor for m/z 153) survey scan, triggering the enhanced product ion (EPI) scan. Spectra were obtained over a range from m/z 100 to 950. The Information Dependent Acquisition criteria were set to select one to two peaks above 1,000 counts, and with an exclusion rule after three occurrences for 30 s with dynamic background subtraction. This method was applied for the characterization/identification of lipid molecular species. Based on the untargeted analysis – product ion and precursor ion analysis of head groups and fatty acyls – a comprehensive list of the multiple reaction monitoring (MRM) transitions was generated (parent R fatty acyl fragment transitions). The quantity of a certain phospholipid molecule from each class was calculated by comparison of its peak area to that of the lipid standard belonging to the relevant class (Supplementary Table 1).

Determination of acylglycerol

Lipids were measured using the same LC–MS/MS model as for the determination of phospholipids. Both triglycerides and diglycerides were detected and identified in the extracted cell samples using single-ion monitoring (SIM) and an EPI method. Then, we prepared MRM scans including parent–daughter pairs of an acylglyceride species reflecting the loss of one fatty acid for TAGs and SIM for DAGs (Supplementary Table 2). For quantification, a standard

curve correlating the peak area to moles of TAGs or DAGs was used with TAG 50:1 as a representative triglyceride and DAG 36:2 as a representative diglyceride. Chromatographic separation was conducted on a C18 column (the same model as mentioned above) that was heated to 40°C. The mobile phases were water (A) and a mixture of acetonitrile:isopropyl alcohol (5:2) with 5 mM ammonium formate and 0.1% formic acid (B). The following mobile phase gradient was used: mobile phase B was increased to 100% from 35% during 4 min; after 11 min, it decreased to 35% over 2 min; the flow rate was set to 0.6 mL min^{-1}. The sample injection volume was 10 μL. The following instrumental settings were applied: curtain gas 25.0, ion spray voltage to 5500 nebulizer gas 60, auxiliary gas 50, and ion source temperature of 600°C, declustering potential to 70.0, entrance potential to 10.0, and polarity to positive. As DAGs and TAGs do not ionize well on their own, an ammonium adduct $[M+NH_4]^+$ needs to be attached to the parent DAG or TAG molecule.

Analysis of sphingolipids

We extracted 100-mg biomass samples with 4 mL ethyl acetate/isopropanol/water mixture (60:30:10, v/v/v) [25]. Qualitative analysis of sphingolipids from evaporated extracts was obtained by examining their mass spectrum using a triple quad mass spectrometer QTRAP 4500 (Sciex) operating in the MRM positive ionization mode as previously described [26]. After reconstitution in 1,000 μL methanol, the samples were injected onto the Agilent 1200 HPLC system (Santa Clara, CA, USA). For the reversed-phase chromatographic analysis, 10 μL of the lipid extract was injected onto a Kinetex C18 column (50 mm × 2.1 mm, particle size: 5 μm; Phenomenex, Torrance, CA, USA). The solvents and gradient elution were identical to that applied for the determination of phospholipids.

Determination of ergosterol

Sterol analysis was undertaken using a QTRAP 3200 (Sciex) mass spectrometer connected to a 1200 series HPLC system. A Kinetex C18 column (50 mm × 2.1 mm, particle size: 5 μm; Phenomenex, Torrance, CA, USA) was used. The solvents were water and methanol, with both containing 5 mM ammonium formate. Analytes were eluted with the following gradient: 40% solvent B from 0 to 1 min, 100% solvent B from 1 to 4 min, 40% solvent B from 4.0 to 4.1 min, 40% solvent B from 4.1 to 6 min with a flow rate of 0.8 mL min^{-1}. The QTRAP instrument was set to the positive ion mode, with an atmospheric pressure chemical ionization (APCI) temperature of 550°C. The monitored MRM pairs were *m/z* 379.3-69.1 and 379.3-81.3.

Test of cell membrane condition

The membrane potential was examined using bis-(1,3-dibutylbarbituric acid)trimethine oxonol (DiBAC4(3)) according to a modified procedure described by Liao et. al [27]. Firstly, 1 mL of the *U. isabellina* culture was transferred into an Eppendorf tube and centrifuged for 5 min at 6,000×*g*. The post-culture medium was removed and the mycelium was suspended in 1 mL MOPS (0.1 M) buffer; 2 μL anionic dye solution (1 mg mL^{-1} in ethanol) was added to the suspension. The mixture was incubated for 5 min in the dark at room temperature. To remove of $DiBAC_4(3)$ residues, the mycelium was washed twice with MOPS buffer.

The membrane fluidity was investigated according to the method by Kuhry et al. [28] with some modifications. Firstly, 1 mL of the culture was transferred into an Eppendorf tube and centrifuged for 5 min at 6,000×*g*. The supernatant was replaced by 1 mL PBS (pH 7). Next, 2 μL 1,6-diphenyl-1,3,5-hexatriene (DPH) solution (6 mM in tetrahydrofuran) was added to the sample, and the mixture was incubated at 37°C (in a water bath) for 45 min. The fungal pellets were washed twice with PBS.

The permeability of the fungal membranes was examined according to the method described by Siewiera et al. [29]. Firstly, 1 mL of the culture was transferred to an Eppendorf tube and centrifuged for 5 min at 6,000 × g. Next, 2 μL propidium iodide (PI) solution (1 mg mL^{-1} in H_2O) was added to the mycelium suspended in 1 mL PBS (pH 7). The sample was kept in the dark for 5 min. The mycelium was washed twice with PBS. Finally, the *U. isabellina* mycelium labeled with one of the pre-specified fluorescent dyes was suspended in 1 mL PBS or MOPS buffer and placed in a 24-well cell culture plate. The fluorescence intensity was evaluated using a FLUOstar Omega (BMG Labtech) spectrofluorometer. The measurement parameters were as follows: λex=485-12 nm, λem=520 nm, gain=1,000 for $DiBAC_4(3)$; λex=355 nm, λem=460 nm, gain=800 for DPH and λex=540-10, λem=630-10, gain=2,000 for propidium iodide. The target was 70%. Additionally, the fluorescence intensity of the supernatant from the labeled sample was measured. The results (calculated through diminishing of sample fluorescence by the background value) were presented as a fluorescence unit per milligram fungal biomass.

ROS determination with the H_2DCFDA technique

The ROS production in the fungal biomass was determined with a cell-permeant (H_2DCFDA; Sigma–Aldrich, Germany) by a method described previously by our team [17]. Fungal biomass samples were centrifuged (5,000×*g*, 5 min). The pellet was washed with 10 mM PBS (pH 7.0), and the mycelium was resuspended in 1 mL PBS (pH 7.0) supplemented with 40 mM H_2DCFDA (dissolved in dimethyl sulfoxide) and incubated for 15 min. Thereafter, the samples were washed with PBS, and images were captured by a LSM510 Meta (Zeiss) using an argon laser (488 nm) and an LP filter set (505–530 nm). The same laser line was used for Nomarski DIC. The results were expressed as a percentage of the green fluorescence area when compared to the total hyphal area.

Extraction and analysis of amino acids

We transferred 100 mg fungal biomass into Eppendorf tubes containing glass beads and 1 mL ethanol (80%) solution in water. Homogenization with FastPrep-24 (MP-Biomedicals) was conducted for 2 min. Next, the sample was centrifuged (2 min, 6,000×*g*) and 50 μL of the supernatant was diluted with deionized water. Fungal amino acid concentrations were determined in duplicates by an aTRAQ Kit for amino acid analysis of physiological fluids (SCIEX) with a QTRAP 4500 (Sciex) mass spectrometer connected to an Eksigent microLC 200 (SCIEX). Detailed analysis was performed according to the manufacturer's instructions.

Data acquisition and statistical analysis

Comparison of the control and 2,4-D–treated mycelium was performed using the mean of three independent biological replicates ± standard error of the means from individual samples. The results were estimated by ANOVA and statistical analyses were performed on three replicates of data obtained from each treatment. The significance ($P<0.05$) of differences was treated statistically by one-, two- or three-way ANOVA. Analysis was performed using the software STATISTICA ver. 13.0 (StatSoft).
