# Supplementary material for: 2,4-dichlorophenoxyacetic acid-induced oxidative stress: Metabolome and membrane modifications in Umbelopsis isabellina, a herbicide degrader
Source: PLoS One. 2018 Jun 22;13(6):e0199677. doi: 10.1371/journal.pone.0199677 (PMC6014680; doi:10.1371/journal.pone.0199677)
Supplement: S1 Table — (PDF) [file pone.0199677.s002.pdf]

S1 Table. Multiple reaction monitoring (MRM) transitions for phospholipids identified in *U. isabellina.*

Lipids	MRM transitions		Lipids	MRM transitions	
PA 16:0/16:1	645.5	253	PS 18:1/18:2	784.5	279
PA 16:0/18:3	669.5	277	PS 18:0/18:3	784.5	277
PA 16:1/18:2	669.5	253	PS 18:1/18:1	786.5	281
PA 16:1/18:1	671.5	253	PG 16:0/18:1	747.5	281
PA 16:0/18:2	671.5	255	PG 18:2/18:3	767.5	279
PA 16:0/18:1	673.5	281	PG 18:1 18:3	769.5	281
PA 18:3/18:3	691.5	277	PC 16:1/16:1	774.5	253
PA 18:2/18:3	693.5	277	PC 16:1/16:0	776.5	253
PA 18:2/18:2	695.5	279	PC 16:0/16:0	778.5	255
PA 18:1/18:3	695.5	281	PC 16:1/18:3	798.5	253
PA 18:1/18:2	697.5	279	PC 16:0/18:3	800.5	277
PA 18:1/18:1	699.5	281	PC 16:1/18:2	800.5	253
PA 18:1/18:0	701.5	281	PC 16:0/18:2	802.5	279
PE 16:1/16:1	686.5	253	PC 16:1/18:1	802.5	253
PE 16:0/16:1	688.5	253	PC 16:0/18:1	804.5	281
PE 16:0/16:0	690.5	255	PC 16:1/18:0	804.5	253
PE 16:1/18:3	710.5	253	PC 16:0/18:0	806.5	255
PE 16:1/18:2	712.5	253	PC 18:3/18:3	822.5	277
PE 16:0/18:3	712.5	277	PC 18:2/18:3	824.5	279
PE 16:1/18:1	714.5	253	PC 18:1/18:3	826.5	277
PE 16:0/18:2	714.5	255	PC 18:2/18:2	826.5	279
PE 16:1/18:0	716.5	253	PC 18:0/18:3	828.5	277
PE 16:0/18:1	716.5	281	PC 18:1/18:2	828.5	279
PE 16:0/18:0	718.5	255	PC 18:0/18:2	830.5	279
PE 18:3/18:3	734.5	277	PC 18:1/18:1	830.5	281
PE 18:2/18:3	736.5	277	PC 18:0/18:1	832.5	281
PE 18:1/18:3	738.5	281	PC 18:0/18:0	834.5	283
PE 18:2/18:2	738.5	279	PI 16:0/16:1	807.5	253
PE 18:0/18:3	740.5	283	PI 16:1/18:3	829.5	253
PE 18:1/18:2	740.5	281	PI 16:1/18:2	831.5	253
PE 18:0/18:2	742.5	283	PI 16:0/18:3	831.5	277

PE 18:1/18:1	742.5	281	PI 16:1/18:1	833.5	281
PE 18:1/18:0	744.5	281	PI 16:0/18:2	833.5	279
PS 16:1/18:2	756.5	253	PI 16:0/18:1	835.5	281
PS 16:0/18:3	756.5	277	PI 16:0/18:0	835.5	283
PS 16:1/18:1	758.5	253	PI 18:3/18:3	853.5	277
PS 16:0/18:2	758.5	255	PI 18:1/18:3	857.5	277
PS 16:0/18:1	760.5	255	PI 18:2/18:2	857.5	279
PS 16:0/18:0	760.5	255	PI 18:1/18:2	859.5	281
PS 18:3/18:3	778.5	277	PI 18:0/18:3	859.5	283
PS 18:3/18:2	780.5	277	PI 18:1/18:1	861.5	281
PS 18:1/18:3	782.5	277	PI 18:0/18:2	861.5	283
PS 18:2/18:2	782.5	279	PI 18:0/18:1	863.5	283
LPC 16:0	494.5	255	PI 18:0/18:0	865.5	283
LPC 18:0	522.5	283	LPC 16:1	492.5	253
LPC 18:2	518.5	279	LPC 18:1	520.5	281
LPC 18:3	516.5	277			
