# Supplementary material for: 2,4-dichlorophenoxyacetic acid-induced oxidative stress: Metabolome and membrane modifications in Umbelopsis isabellina, a herbicide degrader
Source: PLoS One. 2018 Jun 22;13(6):e0199677. doi: 10.1371/journal.pone.0199677 (PMC6014680; doi:10.1371/journal.pone.0199677)
Supplement: S2 Table — (PDF) [file pone.0199677.s003.pdf]

S2 Table. Multiple reaction monitoring (MRM) transitions for triacylglycerols (TAGs) identified in *U. isabellina.*

Lipids	MRM transitions	
TAG 16:0/16:0/16:0	824.7	551.5
TAG 16:0/16:0/18:0	852.8	551.5
TAG 16:0/16:0/18:1	850.8	577.5
TAG 16:0/16:1/18:1	848.8	549.5
TAG 16:0/16:1/18:2	846.8	579.5
TAG 16:0/18:0/18:0	880.8	577.5
TAG 16:1/18:0/18:0	878.8	575.5
TAG 16:0/18:1/18:1	876.8	601.5
TAG 18:1/18:1/16:1	874.8	575.5
TAG 18:2/16:1/18:1	872.8	575.5
TAG 18:2/16:1/18:2	870.8	573.5
TAG 18:3/18:3/16:0	868.8	607.5
TAG 18:0/18:0/18:0	908.8	607.5
TAG 18:1/18:0/18:0	906.8	605.5
TAG 18:1/18:1/18:0	904.8	603.5
TAG 18:1/18:1/18:1	902.8	601.5
TAG 18:2/18:2/18:0	900.8	599.5
TAG 18:1/18:1/18:1	898.8	601.5
TAG 18:3/18:2/18:1	896.8	599.5
TAG 18:3/18:2/18:2	894.8	597.6
TAG 18:2/18:3/18:3	892.8	591.5
TAG 18:3/18:3/18:3	890.8	575.5
