# Supplementary material for: 2,4-dichlorophenoxyacetic acid-induced oxidative stress: Metabolome and membrane modifications in Umbelopsis isabellina, a herbicide degrader
Source: PLoS One. 2018 Jun 22;13(6):e0199677. doi: 10.1371/journal.pone.0199677 (PMC6014680; doi:10.1371/journal.pone.0199677)
Supplement: S1 Fig — (DOCX) [file pone.0199677.s004.docx]

**
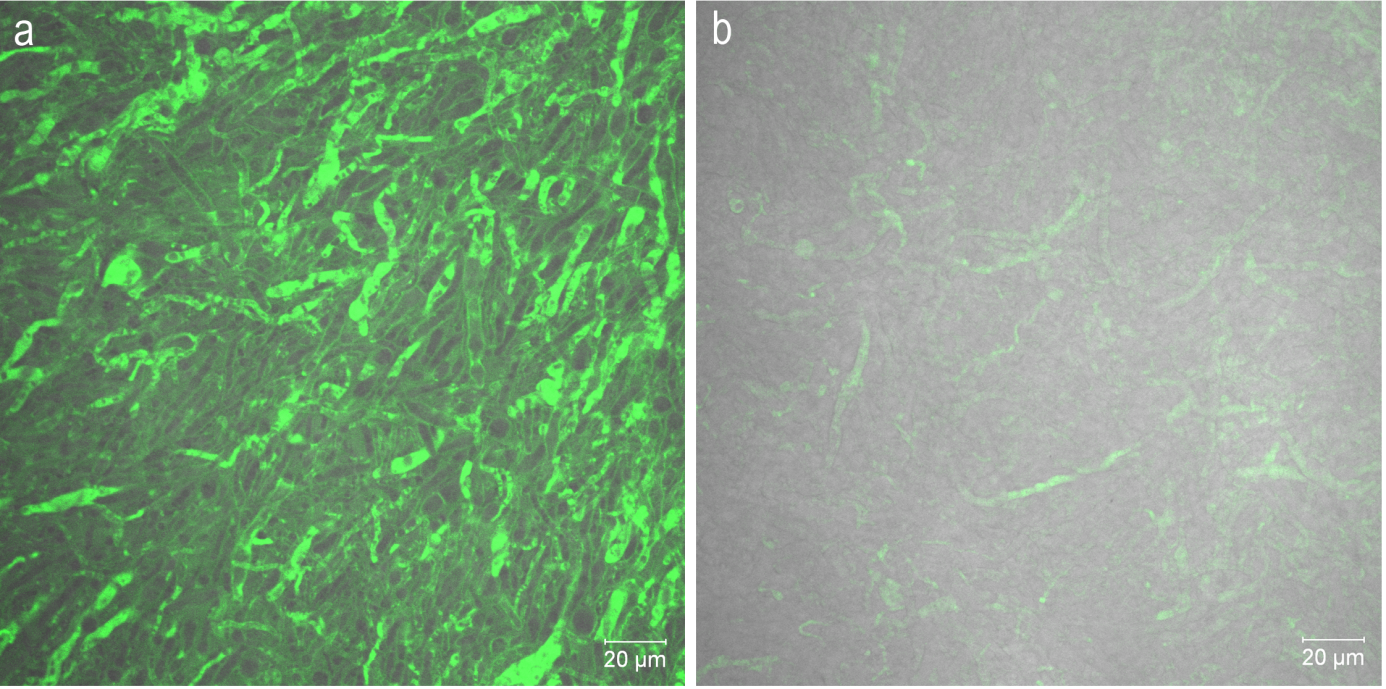
**

**S1 Fig.** ROS (measured with H_2_DCFDA) generated in *U. isabelina* cells incubated with 2,4-D at 24h (a) of incubation in comparison to 24h (b) controls without toxic compound.
