# Supplementary material for: 2,4-dichlorophenoxyacetic acid-induced oxidative stress: Metabolome and membrane modifications in Umbelopsis isabellina, a herbicide degrader
Source: PLoS One. 2018 Jun 22;13(6):e0199677. doi: 10.1371/journal.pone.0199677 (PMC6014680; doi:10.1371/journal.pone.0199677)
Supplement: S2 Fig — (PDF) [file pone.0199677.s005.pdf]

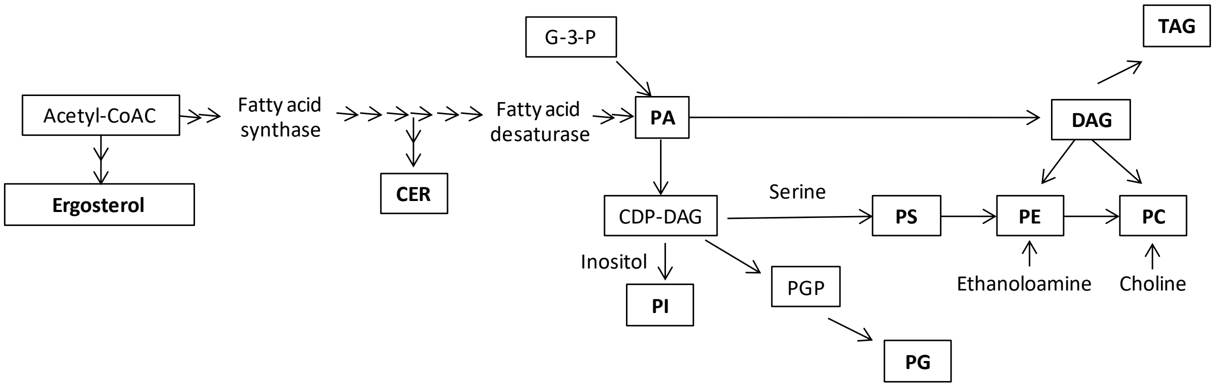

S2 Fig. Hypothetical representation of the lipid metabolism in *U. isabellina*. Boxes in bold indicate lipid classes analyzed in this study The well characterized yeast lipid metabolic pathway was used in the construction of this figure [50]. Abbreviations: G-3-P: glycerol-3-phosphate, Cer: ceramide, PA phosphatidic acid, PE phosphatidylethanolamine, PC phosphatidylcholine, PI phosphatidylinositol, PS phosphatidylserine, LPC lysophosphatidylcholine, TAG- triacylglycerol, DAG-diacylglycerol
