# Supplementary figures and images for: 2,4-dichlorophenoxyacetic acid-induced oxidative stress: Metabolome and membrane modifications in Umbelopsis isabellina, a herbicide degrader
Source: PLoS One. 2018 Jun 22;13(6):e0199677. doi: 10.1371/journal.pone.0199677 (PMC6014680; doi:10.1371/journal.pone.0199677)

A)

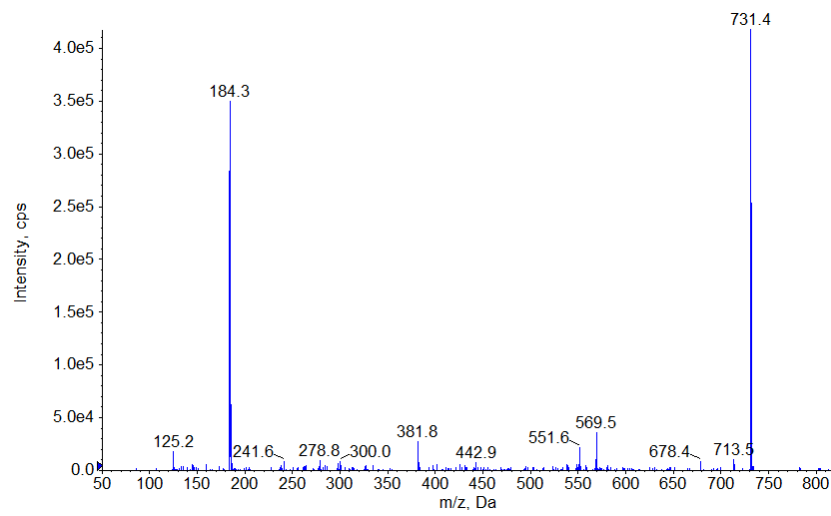

B)

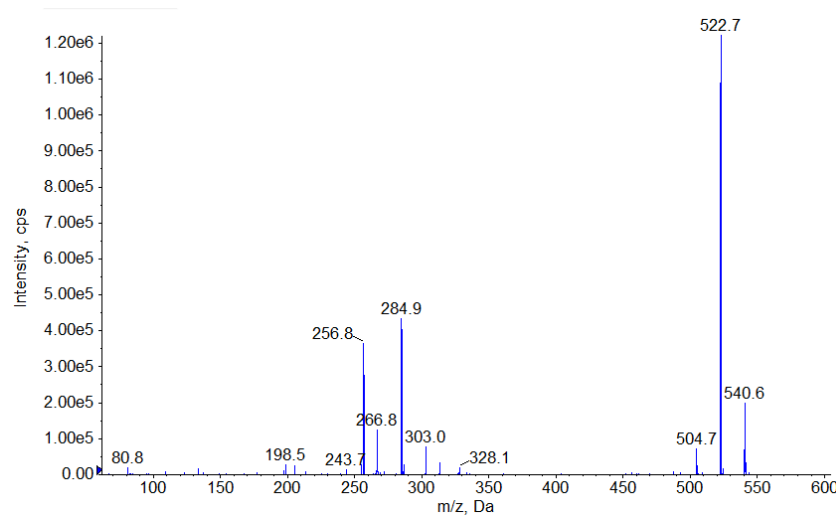

C)

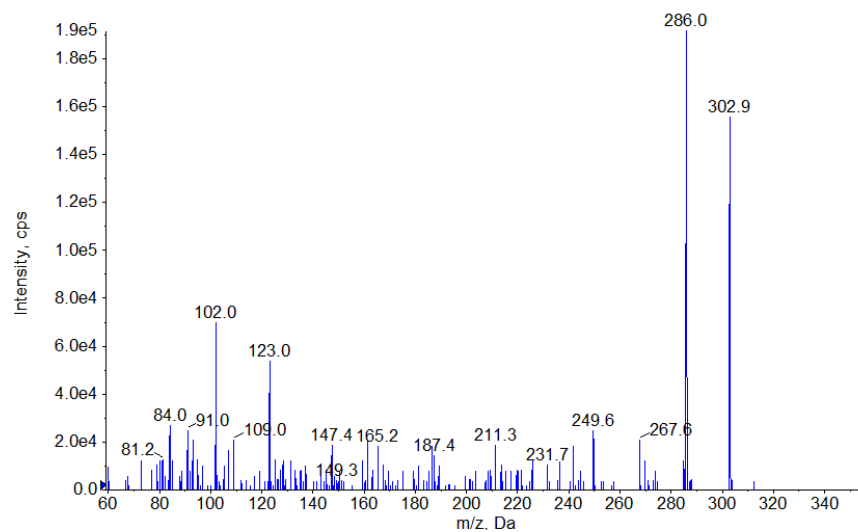

D)

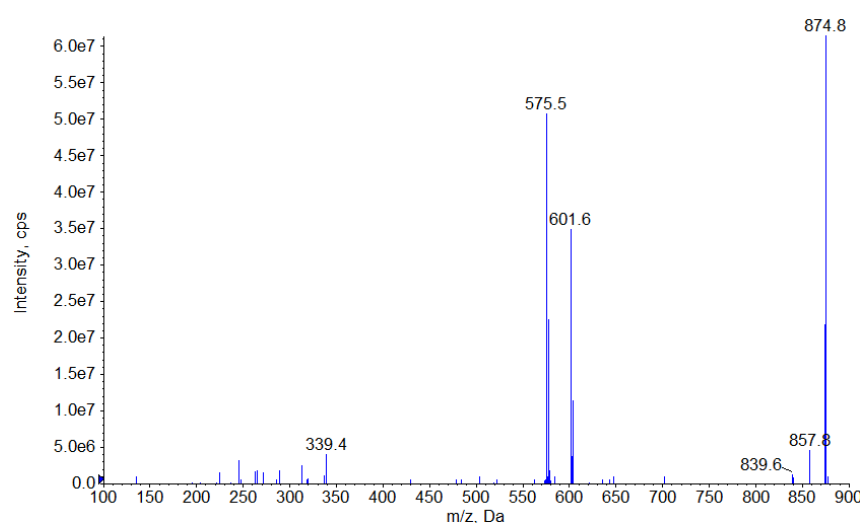

E)

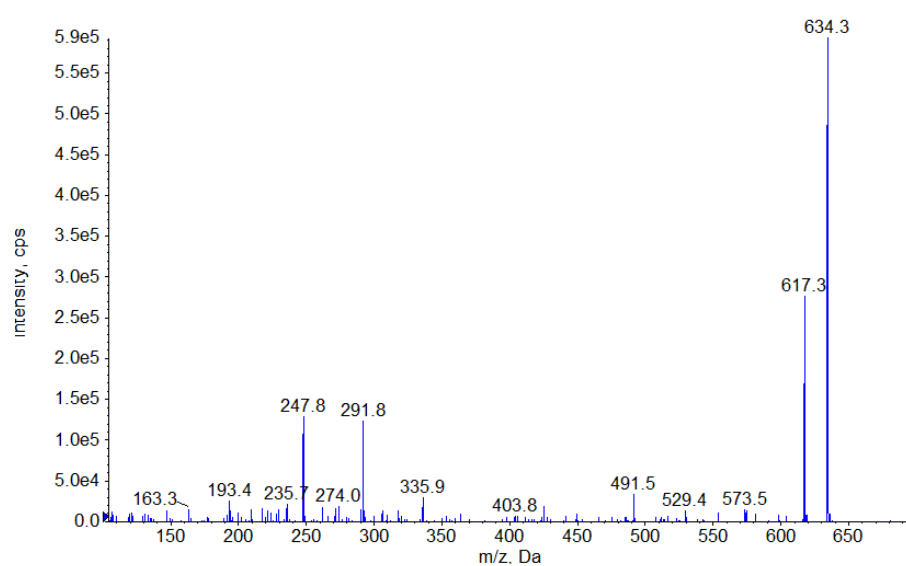

**S3 Fig.** MS/MS spectra of selected lipids. A) C18 SM; B) C16 DhCer; C) C18 dhSpH; D) TAG 52:2

E) DAG 36:4

Supplement: S3 Fig — (PDF) [file pone.0199677.s006.pdf]
